# Automatic Retinal Nerve Fiber Segmentation and the Influence of Intersubject Variability in Ocular Parameters on the Mapping of Retinal Sites to the Pointwise Orientation Angles

**DOI:** 10.3390/jimaging12010047

**Published:** 2026-01-19

**Authors:** Diego Luján Villarreal, Adriana Leticia Vera-Tizatl

**Affiliations:** Departamento de Mecatronica y Biomedica, Escuela de Ingenieria y Ciencias, Instituto Tecnologico y de Estudios Superiores de Monterrey, Monterrey 64700, Mexico; a.l.veratizatl@tec.mx

**Keywords:** structure, function, variability, image segmentation, visual pathways, retinal nerve fiber layer, retinal nerve fiber trajectory, course of axons

## Abstract

The current study investigates the influence of intersubject variability in ocular characteristics on the mapping of visual field (VF) sites to the pointwise directional angles in retinal nerve fiber layer (RNFL) bundle traces. In addition, the performance efficacy on the mapping of VF sites to the optic nerve head (ONH) was compared to ground truth baselines. Fundus photographs of 546 eyes of 546 healthy subjects (with no history of ocular disease or diabetic retinopathy) were enhanced digitally and RNFL bundle traces were segmented based on the Personalized Estimated Segmentation (PES) algorithm’s core technique. A 24-2 VF grid pattern was overlaid onto the photographs in order to relate VF test points to intersecting RNFL bundles. The PES algorithm effectively traced RNFL bundles in fundus images, achieving an average accuracy of 97.6% relative to the Jansonius map through the application of 10th-order Bezier curves. The PES algorithm assembled an average of 4726 RNFL bundles per fundus image based on 4975 sampling points, obtaining a total of 2,580,505 RNFL bundles based on 2,716,321 sampling points. The influence of ocular parameters could be evaluated for 34 out of 52 VF locations. The ONH-fovea angle and the ONH position in relation to the fovea were the most prominent predictors for variations in the mapping of retinal locations to the pointwise directional angle (*p* < 0.001). The variation explained by the model (*R*^2^ value) ranges from 27.6% for visual field location 15 to 77.8% in location 22, with a mean of 56%. Significant individual variability was found in the mapping of VF sites to the ONH, with a mean standard deviation (95% limit) of 16.55° (median 17.68°) for 50 out of 52 VF locations, ranging from less than 1° to 44.05°. The mean entry angles differed from previous baselines by a range of less than 1° to 23.9° (average difference of 10.6° ± 5.53°), and RMSE of 11.94.

## 1. Introduction

For over a century, the intricate relationship between the eye’s structure and function has been of great interest in the context of glaucoma, which was estimated to affect 76 million people worldwide in 2020 [1]. To characterize this connection, several structure-function maps have been developed to describe the anatomy of the optic nerve head (ONH) with the visual field. Early maps have historically been based on scotoma boundaries in the visual field [2], two-dimensional planimetry data of the optic disc [3], insights from primate eyes [4], confocal scanning laser ophthalmoscopy [5], spectral-domain Optical Coherence Tomography (OCT) [6], perimetric sensitivity and en face OCT images [7], and Deep learning training models [8,9,10,11,12]. Recent advances connecting the ONH and visual field (VF) locations in patient populations have been reported. Garway-Heath [13] manually traced visible bundles within the retinal nerve fiber layer (RNFL) from specific sectors of the ONH to clusters of VF test points by superimposing a 24-2 VF grid onto fundus images of 69 eyes of patients with normal tension glaucoma. Lamparter et al. [14] manually tracked RNFL bundle distribution across 100 eyes and fitted by a nonparametric cubic spline model. Jansonius et al. [15,16] constructed a mathematical model derived from manual tracings of RNFL bundles in fundus images of 55 eyes, which enabled additional regions of the central retinal by extrapolation. Denniss et al. [17] recently introduced a novel simulation model that establishes the correspondence between the ONH segments and visual field sites by integrating clinically realistic anatomical parameters.

Structure–function studies are centered to map the spatial relationship concerning the retinal places and ONH segments [13]. Accurate topographical mapping of RNFL bundles at the ONH is crucial for analyzing visual field defects resulting from local optic nerve impairment, which are commonly assessed using OCT imaging [17]. To date, the “ground truth” baselines require manual tracing of visible RNFL bundles to identify their corresponding points of intersection with the ONH. Existing models are often resource-intensive, requiring either excessively expensive ophthalmic equipment to acquire necessary input data [17] or an excessive amount of manual labor for time-consuming procedures to manually [13,14] or electronically [15,16] track RNFL bundles. While ground truth generated models hold significant promise for improvement over currently published models, a notable challenge remains in developing a time- and cost-effective algorithm that balances computational efficiency with the accuracy required for generating precise trajectories. Recent progress in deep learning has demonstrated its ability to autonomously derive task-relevant features from large-scale datasets and capture intricate non-linear associations. Zhiqi et al. [9] introduced a deep learning approach to derive pointwise functional outcomes from segmentation-free three-dimensional (3D) OCT volumes, comparing its performance with two-dimensional (2D) OCT-based depth profiles. Zhiqi et al. [10] used occlusion analysis to establish a generalizable spatial correspondence between ocular structure and function, applying it to a model predicting visual field sensitivities from 3D OCT scans. Park et al. [11] employed Inception V3-based architecture to estimate visual fields from OCT data. Christopher et al. [12] predicted glaucomatous visual field from OCT images by developing a deep learning architecture. Although deep learning models provide end-to-end learning and can autonomously capture complex patterns during training, they have not yet completely or reliably addressed RNFL bundle segmentation, which is essential for establishing structure–function relationships [13], detecting disease and monitoring progression [17], and accurately predicting localized visual field deficits [18]. Deep learning performance is limited by heterogeneity in image acquisition protocols, resolution, and patient demographics. Restricted population diversity undermines evaluative precision, and structural differences in the retina may yield distinct pathological outcomes [19]. Deep learning models struggle to distinguish between tissue layers due to lower light intensity and resolution at greater depths. Segmentation methods are also affected by speckle noise, a common feature in OCT images [20]. VF test results for glaucoma patients are subject to random noise and subjective interpretations, which can further lower prediction accuracy [9]. In anatomically intricate tasks, deep learning methods frequently restrict both clinical utility and interpretative clarity [19,20]. By contrast, advanced image processing methods can directly detect and delineate RNFL bundles, minimizing reliance on large training datasets while enhancing transparency. A key advantage of this method lies in the integration of a structure–function map, enabling patient-precise image segmentation through established anatomical and structural relationships between the ONH and its corresponding VF. This incorporation improves both the precision and biological validity of bundle delineation, providing a clinically meaningful framework for assessing RNFL integrity and yielding stronger and more interpretable results than those generated solely by empirically guided deep learning approaches. The Personalized Estimated Segmentation (PES) algorithm—which is an open-access, automated framework for RNFL bundle tracing developed using image processing methods—holds significant promise to enhance the generation of precise, tailored RNFL bundle maps from accessible inputs [21].

The current study investigates the impact of ocular parameters (ONH position/area, ONH-FO angle, ellipticity ratio) on the mapping of retinal locations to the pointwise directional angle based on the core approach of the PES algorithm—which has been shown to be effective in the segmentation of RNFL bundle traces—in a cohort of 546 eyes from 546 individuals. The performance efficacy on the mapping of VF sites to the entrance angles of RNFL bundles into the ONH was compared quantitatively with respect to the baseline maps generated by Garway-Heath et al. [13], Lamparter et al. [14], and Jansonius et al. [15,16]. In addition, the pointwise angles of orientation were compared quantitatively in regard to the Jansonius map.

## 2. Materials and Methods

### 2.1. Subjects and Fundus Photographs

Digitized retinal photographs from 546 eyes of 546 healthy subjects (with no history of ocular disease or diabetic retinopathy) were obtained from the MESSIDOR database [22]. The dataset comprises 1200 8-bit RGB retinal photographs with resolutions of 1440 × 960, 2240 × 1488, or 2304 × 1536 pixels, acquired by a 3CCD colored video camera attached to a Topcon TRC NW6 non-mydriatic fundus camera (Topcon Corporation, Tokyo, Japan) with a FOV of 45°. An accompanying Excel file provides visual impaired diagnoses for each image, and only images from healthy fundi were selected for the present study. The collection of images is reported to adhere to relevant ethical regulations for human subject research, as accepted by the respective Ethics Committee.

### 2.2. Retinal Nerve Fiber Bundle Assessment

The PES image analysis algorithm as described earlier [21] is used for segmenting and aligning the retinal nerve fiber (RNF) layer bundles from fundus images. The process is broken down into four main stages. First, the algorithm isolates the main circular region of the fundus image by creating a binary mask to remove unwanted text and noise, ensuring only the region of interest (ROI) is analyzed. Second, noise is reduced using a median filter and illumination is corrected using a sigmoid function for adjusting pixel intensity and improving low-light images. Blood vessels are removed using the Contrast-Limited Adaptive Histogram Equalization (CLAHE) [23] to boost contrast, followed by the Hessian-based multiscale Frangi vesselness (HFV) filter [24] to identify and remove the tubular structures of the vessels. CLAHE enhances local contrast until the regional histogram approximates a uniformly distributed curve, while constraining the enhancement to avoid amplification of image noise. HFV filtering is a targeted feature improvement technique designed to segment and evaluate elongated vessel-like formations. This process relies on the Hessian matrix, whose localized derivatives of the 2nd order quantify the curvature of the intensity profile. By characterizing variations in the image gradient, these measures are essential for detecting distinct geometric patterns. Third, the maximum-minimum modulation (MM) algorithm [21] is used for normalizing pixel intensities and increasing luminance differences between RNF segments and their surroundings, making their borders more prominent. The RNF segments and the vessel extraction are then binarized via the Otsu technique and combined via image subtraction to yield a clean RNF map. Fourth, the algorithm aligns the segmented RNFs to create continuous bundles. The directional angle (seen from the ONH center) of individual RNF segments—termed “pointwise RNF extraction”—is identified by using a modified Garway-Heath map [13] and the Jansonius map [15,16]. False positives (non-RNF structures) are eliminated by filtering out segments whose orientation do not fall within the expected ranges. The average angular range for fine-tuning RNF orientations exhibited considerable variability (70° ± 21°) [21]. The algorithm assembles complete bundles by connecting neighboring RNF extractions, which are subsequently modeled with a *n*th-order Bezier curve to capture their spatial progression from sectors at the retina to the ONH. Entrance angles for each bundle were determined by the crossing of bundle maps with the ONH boundary. Performance metrics, including the effective percentage and root mean square error (RMSE), were calculated as described in [21]. A 45° OCT field of view was employed. Retinal photographs were incorporated only if the fovea and/or ONH could be precisely located, and bundles required at least eleven marker points to fit a 10th-order Bezier curve. The PES algorithm processes images of approximately 8.1 megapixels (2330 × 3500) in under 45 s, performing a fixed sequence of operations for feature computation, including segmentation, Bezier curve fitting, structure–function mapping, and image rendering.

**Figure 1:**
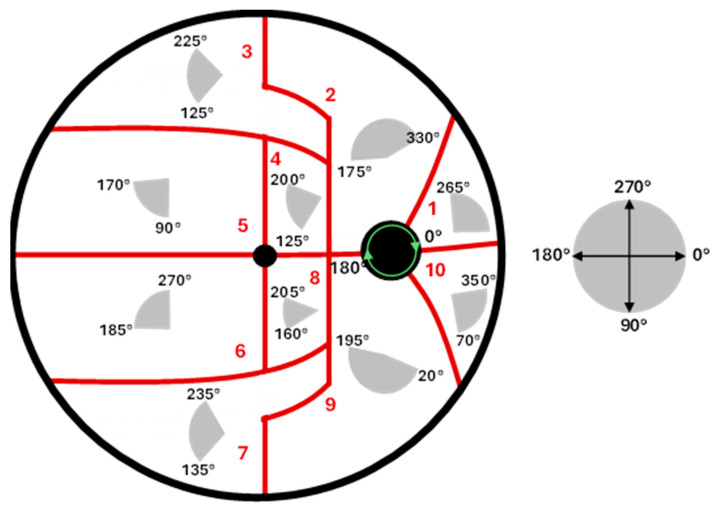
Representative fundus image and the corresponding orientation map. The FOV of the fundus image was separated into 10 regions. For each, the directional angles were computed as seen in [21]. The angle convention indicated by the green arrow is defined around the ONH boundary.

Figure 1 shows the map of the directional angles computed as seen in [21]. As a summary, the FOV of the fundus image was separated into 10 regions. The Jansonius map was traced with a 1-degree step, and the directional angles were computed per sample point as seen from the ONH center. Angular ranges for each region were defined by identifying the maximum and minimum angles present within the respective area. Model performance was subsequently refined by iteratively adjusting the boundaries for each set of angles, employing a coarse sampling interval to maintain computational efficiency. Directional angle fitting was conducted using 150 of the 300 available fundus images. The configuration that produced the highest mean effective percentage was retained for further analysis (see Equation (1) in [21] for the calculation of the effective percentage).

### 2.3. General Procedure for the Visual Field Map

A 24-2 Humphrey Field Analyzer grid (VF test points 6° apart) was properly scaled and aligned within the FOV fundus image centered on the FO. The ONH position relative to the FO was recorded, with 0° assigned to the nasal margin (3 o’clock, right eye) and angles measured clockwise. The result obtained from the RNFL bundles were required to lie within 0.86° of a visual field point in order to comply with being twice the diameter of the Goldman Size III stimulus. Normalization and rotation were used by bringing the centers of the ONH and FO into line as have been recently described by [13,14,15,16]. This general procedure establishes the foundational framework, which then diverges into two separate analytical paths corresponding to the first and second statistical tests, respectively, as detailed in the following section.

### 2.4. Methodology for Statistical Analyses

Three statistical tests were performed in this study. In the first test, the individual deviations were studied by determining the effect of the next parameters possibly related with the bundle traces: ONH area, ONH position, disk–fovea angle, and ellipticity ratio. The dependent variable is the mean pointwise directional angle at individual visual field location. To accomplish this, the general visual field mapping procedure used the result obtained from the RNFL bundles to compute their pointwise directional angles relative to the center of the ONH. The directional angles were mapped along with the VF sites onto a pixel image with the FO centered.

Image dilation was applied exclusively to the pointwise angle representations to account for pixel spacing, after which their crossing with each superimposed VF location were computed. To reflect the inverse mapping between retina and visual field, superior points were assigned their pointwise directional angles corresponding to the mirrored inferior locations, and vice versa. For each visual field point, the maximum number of crossings was determined by the number of pixels along its circumference at the selected image resolution. To preserve continuity across the 0°/360° boundary, sector ranges with variance greater than 180° were adjusted by subtracting 360°. The Akaike information criterion (AIC) [25] in a forward stepwise regression was chosen as an optimal model for predicting the pointwise directional angle. The AIC selects the optimal model from a group of candidates by evaluating the model quality and minimizing information loss using the fewest possible parameters. The resulting model, therefore, identifies the most influential parameters for predicting the intersecting pointwise directional angles. This study evaluates the curvature-related analysis of the RNFL bundle information at each visual field point, providing a detailed, granular analysis of data by examining angle deviations at each individual location and focusing on the specific behavior of the associated parameters at discrete points. Statistical significance in the regression was defined as *p* < 0.001. A quantitative comparison of the individual variation for given VF locations was carried out between Lamparter et al. [14] and the current study. A comparison was also performed on the statistically significant candidates of ocular parameters for the visual field points.

In the second test, the performance efficacy on the mapping of VF sites to the entrance angles of traces into the ONH was compared quantitatively with respect to the baseline maps generated by Garway-Heath et al. [13], Lamparter et al. [14], and Jansonius et al. [15,16]. To accomplish this, the general visual field mapping procedure used the result obtained from the RNFL bundles which were mapped along with the VF sites onto a pixel image with the FO centered. Image dilation was performed only to the RNFL bundles to mitigate pixel spacing and their crossing with each superimposed VF point were subsequently computed. To reflect the inverse mapping between retina and visual field, superior points were assigned their ONH entry positions corresponding to the mirrored inferior locations, and vice versa. To preserve continuity across the 0°/360° boundary, sector ranges with variance greater than 180° were adjusted by subtracting 360°. The mean entrance angles of the baseline maps were averaged and compared quantitatively with the current study. The RMSE of the entrance angles into the ONH was calculated as recently defined by [21]. This quantitative analysis evaluates the efficiency of bundle trace convergence at the ONH by evaluating the spatial association among specific ONH sections and their corresponding bundle trajectory.

In the third test, the pointwise angles of orientation were compared quantitatively with respect to the Jansonius map using the RMSE, the mean, and the standard deviation of the differences, as defined by [21]. This localized angular difference examines the efficacy of the curvature-associated evaluation of bundle traces. The statistical analyses were carried out in Statistics and Machine Learning Toolbox^TM^ (Version 24.2) in the MATLAB environment (R2024b Update 6, version 24.2, 64-bit, Windows; The MathWorks, Inc., Natick, MA, USA).

### 2.5. Ocular Parameters

The influence of multiple ocular factors on the local directional angle at each point was investigated. Parameter estimation followed the procedure described in [21] and was adopted here without modification. Fundus images were excluded if the FO or the ONH could not be precisely identified. For the ONH center, the sharpest image of the ONH ROI contour from two independent algorithms was used for identifying five points along the boundary. An elliptical closed contour was then fitted using the Gal [26] algorithm with a Least Squares approach, from which the ONH center and its horizontal and vertical radii were calculated. The ONH area was estimated as the product of the radii and π, expressed in degrees. Similarly, the FO center was determined from the sharpest ROI contour derived from the two separate algorithms. These separate algorithms enhanced the FO- and ONH-ROI to specifically target contour edges for accurate identification. The location of the ONH relative to the FO is defined by its horizontal and vertical distances. The horizontal distance is the separation along the *x*-axis between the center of the ONH and the FO, while the vertical distance is the separation along the *y*-axis between the ONH center and the horizontal meridian (measured in degrees). The ellipticity ratio was defined as the ratio of the minor to major ONH radius. The disk–fovea angle was calculated as the inverse tangent of the vertical-to-horizontal radius ratio (ONH_y_ and ONH_x_, respectively), expressed in degrees

Table 1 provides an overview of the parameters associated between individual deviations. ONH_x_ and ONH_y_ ranged from 11.57 to 21.11 degrees and from 0.04 to 6.01 degrees, respectively. A wide-ranging variation was also present for the ONH-FO angle, the ellipticity ratio and the ONH area.

## 3. Results

Digitized retinal photographs from 546 eyes of 546 healthy subjects (with no history of ocular disease or diabetic retinopathy) were included in this study. The PES algorithm assembled an average of 4726 RNFL bundles per fundus image based on 4975 sampling points (or pointwise RNF extractions), obtaining a total of 2,580,505 RNFL bundles based on 2,716,321 sampling points. On average across all retinal photographs, the algorithm was effective 97.6% and worked well with those of low resolution. No investigation was performed on the blind spot, which is represented by the two field points above and below the ONH center.

**Figure 2:**
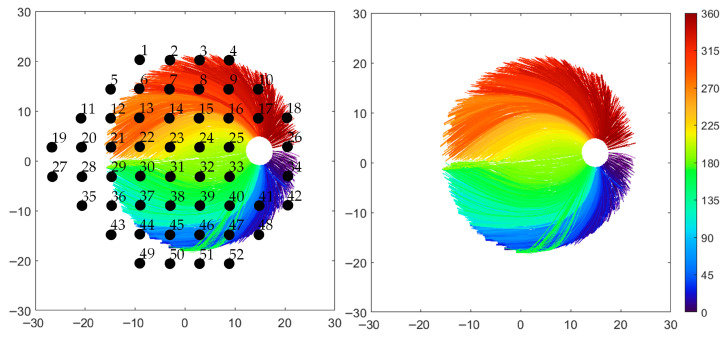
(**Left**): RNFL bundle distribution comprising 11,031 bundles derived from approximately 11,611 pointwise extractions obtained from two subjects. Fifty-two visual field points are aligned with the bundle map, with superior field locations assigned to the ONH entry of their mirrored inferior counterparts, and vice versa. (**Right**): RNFL bundle distribution.

Figure 2 shows the distribution of RNFL bundles and their corresponding visual field positions. 11,031 RNFL bundles, based on roughly 11,611 sample points were derived from only two subjects. 52 visual field sites were aligned within the fundus image FOV. For reporting, the superior points were designated the ONH entry positions corresponding to mirrored inferior locations, and vice versa in order to reproduce the inverse mapping between retina and visual field.

**Figure 3:**
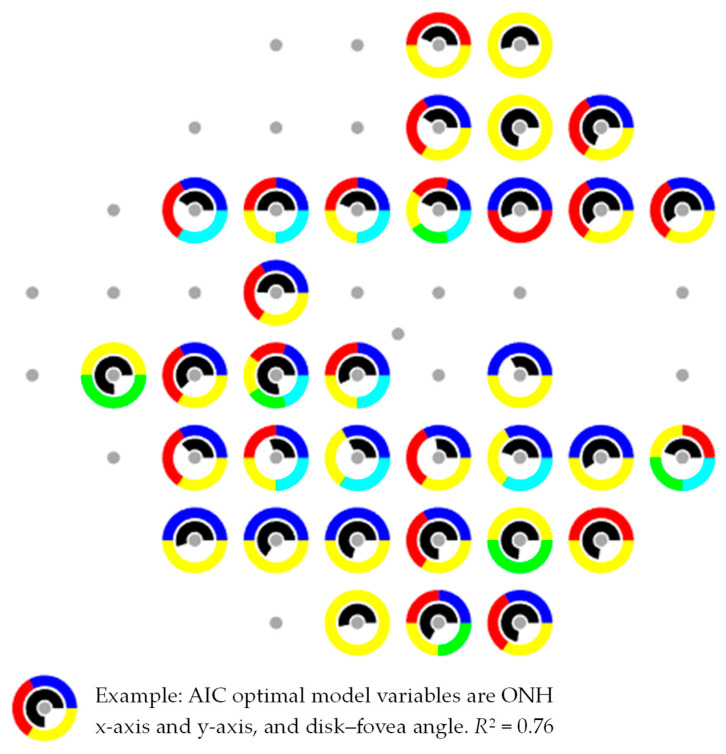
Relevant ocular parameters chosen as the AIC optimum model. The scattered gray dots represent different locations in the visual field. The colored sections within a circle correspond to the most influential variables per visual field point for predicting the intersecting pointwise directional angles: OD area (cyan), ONH position (blue, *x*-axis) and (red, *y*-axis), disk–fovea angle (yellow), and ellipticity ratio (green) for each of the 34 visual field locations. The arc-length of the colored sections does not represent the weight of contribution of the influential variables. Black section represents the variation explained (*R*^2^ value). The arc length of the black section is proportional to the *R*^2^ value. The results represent statistical significance (*p* < 0.001).

Figure 3 shows the relevant ocular parameters chosen by the AIC optimum model. The scattered gray dots represent different locations in the visual field. The colored sections within a circle correspond to the most influential variables per visual field point for predicting the intersecting pointwise directional angles: ONH area (cyan), ONH position (blue, *x*-axis) and (red, *y*-axis), disk–fovea angle (yellow), and ellipticity ratio (green) for each of the 34 visual field locations. The arc-length of the colored sections does not represent the weight of contribution of the influential variables. Black section denotes the variation explained (*R*^2^ value). The arc length of the black section is proportional to the *R*^2^ value. The results represent statistical significance (*p* < 0.001).

Table 2 shows the mean ± SD (95% limit) of the directional angles at each visual field point (2nd and 3rd row, respectively), in degrees. The directional angles from individual RNFL bundle intersect a certain field point at a very variable location (9.5 of mean standard deviation (95% limit) of all locations). The percentage of individual variation explained by the optimum AIC model (PV, 4th row) is also shown. Thirty-four VF locations entered in the current study. The variation explained by the model (*R*^2^ value) ranges from 27.6% for visual field location 15 to 77.8% in location 22, with a mean of 56%.

**Figure 4:**
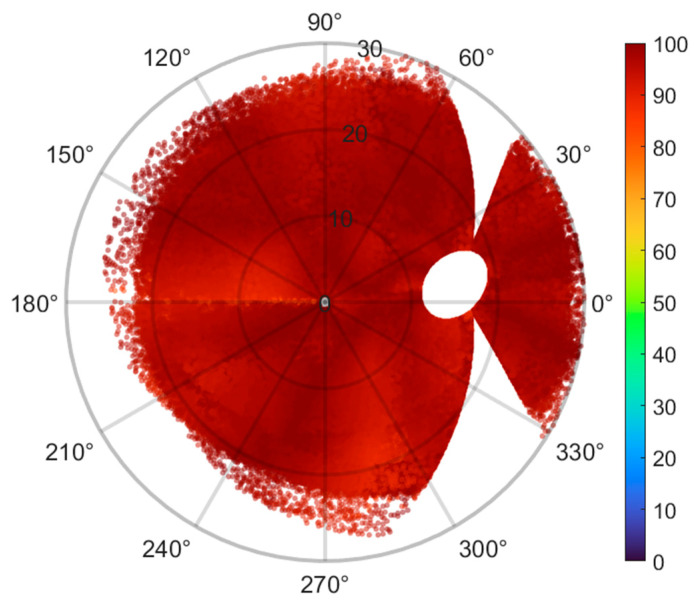
Effective percentage per extraction for the 546 fundus images from the MESSIDOR database. Each fundus image was superimposed onto the Jansonius map via translation for centering the FO, followed by zooming and rotation for aligning the ONH centers at an eccentricity of 15°, 2° above the horizontal meridian. The algorithm extracted a total number of 2,716,321 sampling points seen as scatter dots. The singularity along the horizontal line temporal to the fovea can be attributed to the difficulty in tracing RNFL bundles near the raphe.

Figure 4 shows the effective percentage per extraction for the 546 fundus images from the MESSIDOR database.

Each fundus image was aligned with the Jansonius map by translating to center the FO, then scaled and rotated to align the ONH centers at 15° eccentricity, 2° over the horizontal meridian. The effective percentage was computed as recently described by [21]. The algorithm extracted a total number of 2,716,321 sampling points seen as scatter dots.

The singularity along the horizontal line temporal to the fovea can be attributed to the difficulty in tracing RNFL bundles near the raphe.

Table 3 shows the optimal AIC model for each visual field location. It is worth noting that parameters defining the ONH–FO angle and the ONH position concerning the FO were statistically significant for practically the entire visual field. Variability in ONH area accounted for variation in nine visual field locations, followed by variation in the ellipticity ratio that influenced the mapping in six locations.

**Figure 5:**
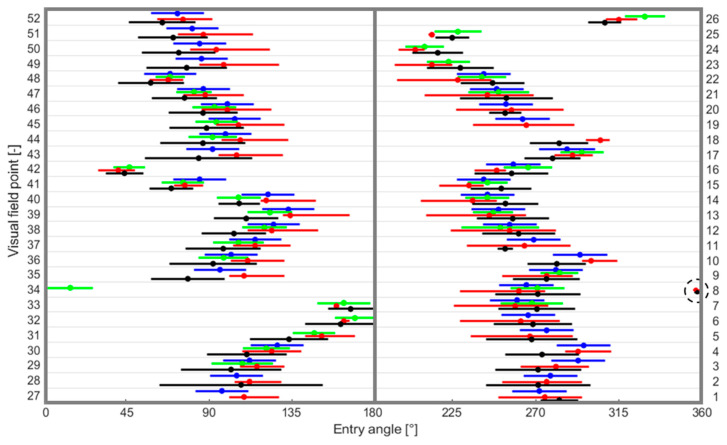
Quantitative comparison quantitative comparison of the ONH segments derived in this study with those documented by Garway-Heath et al. [13], Lamparter et al. [14], and Jansonius et al. [15,16]. Each column (or plot) corresponds to a hemifield from the Garway-Heath map, with rows indicating the VF locations. Blue curves indicate the mean and 95% confidence limits from [13], while red curves show the predicted ONH mean and sector limits reported by [15,16]. Green curves denote the mean and standard deviation reported by [14], while the black curves indicate the mean with 95% limits from the present study. The ONH result within the dashed black circle corresponds to the 34th visual field point.

Figure 5 displays a quantitative comparison of the ONH segments derived in this study with those documented by Garway-Heath et al. [13], Lamparter et al. [14], and Jansonius et al. [15,16]. Each column (or plot) corresponds to a hemifield from the Garway-Heath map, with rows indicating the VF locations. Blue curves indicate the mean and 95% confidence limits from [13], while red curves show the predicted ONH mean and sector limits reported by [15,16]. Green curves denote the mean and standard deviation reported by [14], while the black curves indicate the mean with 95% limits from the present study. The ONH result within the dashed black circle corresponds to the 34th visual field point.

## 4. Discussion

### 4.1. Agreements with Existing RNFL Bundle Paths

The current results showed strong quantitative and qualitative agreement with the Jansonius map across most regions (Figure 4). Average mean variation in directional angle across the retinal photographs was 10.37° ± 0.81°, with average RMSE of 13.3 ± 1.05. These improved results related to previously reported [21] (average mean difference 11.01 ± 1.25, with average RMSE 13.82 ± 1.55) are attributed to the increase in the Bezier curve order to 10th, allowing the RNFL bundles to arise from the bordering temporal regions over or under the macula, entering the ONH along a clearly defined arcuate trajectory within the temporal upper and lower quadrants, see Figure 2.

Bezier fitting curves were employed as a uniform approach, accommodating curve traces that violate the criteria of the vertical line test—where the curve fails to produce a unique value for each x-coordinate—commonly observed in fibers entering the ONH inferonasal region, which pose particular challenges for curve fitting. This issue has been documented in Figure 2a of [15], Figure 3 of [16], and Figure 4a,b of [27]. This quantitative and qualitative agreement remains valid, except for a discordance observed along the horizontal line temporal to the FO, as noted in earlier publications [21], refer to Figure 4. This discordance can be attributed to the difficulty in tracing RNFL bundles near the raphe, where it is challenging to determine which fibers originate from and which simply pass over that area of interest. Lamparter et al. [14] and Jansonius et al. [15,16,27] reported that RNFL bundles near the horizontal meridian temporal to the FO are generally not manually traced, except for those crossing at approximately 180° minus the fovea–disc angle in the superior hemifield [14,15] or at 180° plus the fovea–disc angle in the inferior hemifield [27]. This pattern corresponds to the discrepancies observed between the present findings and previous reports (Figure 4). An additional source of discordance may be related from limitations of the algorithm [21], which does not incorporate structural parameters of the optic disc, such as tilt angle, axial length, spherical equivalent, or other unaccounted factors. These factors could potentially provide insights into accurate mapping in certain eyes. Additionally, RNFL bundle organization may be influenced by factors such as axial elongation, which amplifies the ONH-to-FO distance in myopic eyes [28], differences in ganglion cell quantity and distribution, the arrangement and depth of retinal blood vessels, and developmental growth signals [29,30]

### 4.2. Intersubject Variability in Ocular Parameters for Pointwise Directional Angles (AIC Test)

This study examined the influence of variation in ocular parameters on the mapping of visual field sites to the pointwise directional angle. The maximum number of crossings for each visual field point is 500 determined by the chosen image resolution of 2300 × 3500 pixels. A total of 34 field points were included in the statistical analysis (*p* < 0.001). According to the current findings, the anatomical variations significantly affect the relationship between certain visual field locations and the pointwise directional angle, as depicted in Figure 3 and Table 3. For some sites, a single or a couple of ocular parameters were found to influence this relationship (e.g., sites 2, 5, 6, 7, 9, 10, 14, 17, 20, 25, 40, 47, 51 and 52), while others were found to be significantly impacted from multiple different ocular factors (up to four parameters, predominantly for sites 3, 13, 18, 22, 23, 30, 36, 37, 38, and 39). The most prominent parameters were the ONH position (*x*-axis, 26 cases), (*y*-axis, 22 cases), and the ONH–FO angle (32 cases). Ellipticity ratio (6 cases) and the ONH area (9 cases) were the next most strongly associated. Prior research has revealed the role of ONH location as a characteristic that explains between-subject variability [13,14,17], and individual differences in ocular anatomy influence how visual field locations are mapped. Denniss et al. noted that the structure–function relationship is significantly influenced by the ONH’s position with respect to the fovea, axial length, and horizontal and vertical ONH diameters. Lamparter et al. reported that intersubject variability on entrance angle into the ONH was impacted by the latitude and longitude distance and disk area of the ONH, followed by variations in orientation, ellipticity ratio and ONH tilt. Garway-Heath et al. reported that the position of the ONH was discovered to be a factor in clarifying intersubject variation. In the current study, an agreement of ocular parameters was found to have a significant impact on the visual field relationship. In agreement with Lamparter et al., Denniss et al., and Garway-Heath et al., the key determinants of the variations observed in this study are the ONH–FO angle and ONH position, followed by the ONH area and ellipticity ratio, which are in good agreement. The variation explained by the model (*R*^2^ value) ranges from 27.6% for visual field location 15 to 77.8% in location 22, with a mean of 56%, which have similar results to Lamparter et al. of 60%, see Table 3. The reason for this disparity may be the quantity of ocular factors that were absent from this study but included in Lamparter’s model, such as the axial length, spherical equivalent, ONH tilt, and ONH orientation. According to the AIC analysis, the results offer an understanding of the factors impacting the pointwise directional angle on visual field sites since explanatory power was substantial obtained, accounting for a significant amount of the explained variation. Even though the model accounts for a significant amount of the variance, 44% of the variability cannot be explained. That means the dependent variable is also influenced by other factors that are not included in the current model. These unmeasured characteristics, such the axial length, should be investigated in future studies to improve our understanding. Numerous studies provide evidence that the RNFL bundles, especially the papillomacular bundle, lengthen when axial length increases, especially in myopic eyes [31,32]. Since the axial length varies by 23.4 ± 1.04 mm, with a range of 20.29 to 28.68 mm [33], any change in its spatial connection has a direct impact on the papillomacular bundles’ length, curvature, and overall movement [31]. Because of its extremely straight trajectory, the papillomacular bundle is more susceptible, which may limit its ability to adapt to the widening ONH–FO gap. On the other hand, the more peripheral nerve fibers that are connected to nontemporal rim sectors have a more arcuate course, which may allow them to straighten out more efficiently [32]. As was previously mentioned, one drawback of the fundus images is that the axial length is inaccessible and, as a result, is not utilized in this investigation. As a result, rigorous evaluation of these noteworthy effects’ clinical importance is necessary.

### 4.3. Quantitative Performance of the Visual Field Map into the ONH

This study examined a quantitative comparison to compare the mean entrance angles of trajectories at each visual field point into the ONH. The maximum number of crossings for each visual field point is 500 determined by the image resolution of 2300 × 3500 pixels. The map shown in this study aligns well with earlier results, as seen in Figure 5. Jansonius et al. [16] reported an average 8.8° (ranging from under 1° to 18°, median 7.3°) standard deviation of the entrance angle into the ONH, computed as a quarter of the 95% limits. Lamparter et al. observed variability among individuals at the ONH entrance point, with an average 12.4° of standard deviation. Garway-Heath et al. found a mean standard deviation of 7.2° at the ONH circumference. In this study, there was a large intersubject variability in RNFL bundle courses into the ONH (average standard deviation, including the 95% limit calculated as ± two times the standard deviation), was 16.55° (median 17.68°) for 50 of 52 visual field locations, missing only data for 19h and 27h. The results indicate that the standard deviation varies significantly based on the visual field test point, ranging from under 1° to 44.05°. There was a greater individual variability in ONH entry position in this study compared with the previous report [21] (mean standard deviation (95% limit) 13.5°). One possible justification is the wide variation in disc–fovea angles among the subjects (7.07° ± 3.64°, range −2.6° to 19.76°) and the similarly to the variability in ONH positions, which account for much of the variance in structure–function maps [13,17] (Table 1). The entrance angles found in this study differed from the average of previous baseline studies [13,14,16] by 1° to 23.9° (average difference of 10.6° ± 5.53°), and RMSE of 11.94. To enable a consistent evaluation, the data point at the 34th VF site was omitted since the inconsistencies at the 0°/360° boundary. Despite the numerical differences in the average entrance angles, the overlapping standard deviation ranges show that the various groups share a common range of values due to natural variation. These results are promising for developing personalized structure-function tests, which could improve the accuracy of detecting and monitoring glaucoma progression.

### 4.4. Literature Review

Trajectories of RNFL bundles have been described in a number of prior articles. The studies were based on postmortem human eyes [34], monkey histology [35], fundus photography [13] correlations between perimetric sensitivities and optic disk anatomy by Ferreras et al. [36] and Turpin et al. [5], and optical coherence tomography by Garvin et al. [37]. Similar to the trajectory path indicated by the Jansonius map, Wigelius et al. [38] provided a numerical framework that delimited an implicit solution. Weber and Ulrich [2] developed an RNFL bundle map according to scotoma limits in bundle impairs. Even though it was less accurate and thorough, this map showed a pattern that was virtually the same as the idea the Jansonius map explained. A mathematical framework explaining the normal trajectory and variances in RNFL bundle traces was created by Jansonius et al. [15,16]. An RNFL optical texture analysis (ROTA) technique was introduced by Leung et al. [39]. It combines RNFL thickness and reflectance information from OCT scans to reveal the optical textural characteristics of RNFL bundles through a sequence of non-linear transformations. Dennis et al. [17] proposed an algorithm that divided the ONH head into sectors and the retina into a grid of elements to create an anatomically configurable fiber mapping method.

### 4.5. Novelty and Specific Advantages of the PES Algorithm

The PES algorithm addresses the challenge of accurately segmenting the RNFL bundle trajectories in the human retina while avoiding the common limitations of deep learning methods [21]. The advantages include advanced image processing strategies to inherently detect and delineate RNFL bundles, thereby incorporating the structure–function mapping framework with highly explainable decision logic process, and directing patient-precise trace delineation by integrating established anatomical–structure correlations between the ONH and its associated visual field. This incorporation improves both the precision and biological validity of segmentation, providing a clinically meaningful framework for assessing RNFL integrity. Instead of seeking long RNFL bundle traces from the most prominent and visible region near the ONH toward the retinal periphery, the PES algorithm introduces a novel strategy by identifying localized patches of bundle trajectories with angle orientations that satisfy predefined angular criteria specific to distinct regions within the fundus FOV. The criteria were defined using the directional angle of the Jansonius map relative to the ONH center. Regions from a modified Garway-Heath map [13] were aligned with the bundle traces, and angular thresholds were set by the minimum and maximum values within each region. Algorithm performance was optimized by iteratively adjusting these limits and computing the effective percentage on a test set of 150 fundus images, as described in Equation (1) of [21]. This angular criterion reduces false positives by excluding non-RNFL structures. Bundle trajectories were then modeled with a *n*th-order Bezier function by linking the nearest pointwise extractions based on the directional angle. Bezier curves provided a uniform solution, including cases that failed the vertical line test most often observed in fibers entering the inferonasal ONH, which are particularly difficult to fit. Similar singularities have been reported in Figure 2a of [15], Figure 3 of [16], and Figure 4a,b of [27].

The overall precision of the PES algorithm arises from a sequence of three processing phases. In the first phase, CLAHE algorithm suppresses excessive noise propagation while enhancing local contrast, followed by the HFV filter to amplify and detect vessel-like formations, a critical step for delineating curvilinear RNFL patterns. In the second phase, the MM algorithm identifies local intensity extrema within a region, thereby assessing edge sharpness and isolating bundle sections. Through this redistribution of pixel intensities, luminance differences between RNFL extractions and their surrounding background are accentuated. The third phase enforces orientation consistency of RNFL extractions by constraining them to predefined angular sets based on the Jansonius map and a modified Garway-Heath map [13]. Employing an algorithm to trace RNFL bundles enhances the identification of local optic nerve impairment and associated visual field damages, offering the potential to substantially enhance clinical assessment and management of conditions such as glaucoma and visual field impairment.

### 4.6. Limitations

Several unaccounted-for factors may influence mapping accuracy in certain eyes. Structural parameters of the optic disc—such as tilt angle, axial length (typically measured by partial coherence interferometry), spherical equivalent, and ONH orientation (evaluated with Spectralis HRA reflectance imaging) [14]—were not considered. Lamparter et al. reported that axial length and spherical equivalent affected mapping at 9 of 33 visual field locations. In the present study, the impact of these variables could not be assessed because the retinal photographs were sourced from protected-access datasets without biometric data on axial length or refraction. Additional deviations may arise from other influences on RNFL architecture, including developmental variability, vessel depth, and the number and distribution of ganglion cells [29,30]. Variability in ocular parameters across subjects may be strongly influenced by genetics. About 80% of the diversity in the ONH appearance, particularly in its diameter (disc size) and general shape shapes, is heritable, with nonshared environmental influences accounting for 20% of the variability [40,41,42]. The substantial noise that characterizes fundus image associations is a major barrier to combining imaging data with structural maps. This noise likely has multiple causes, such as intersubject variations in the association between the ONH and retinal regions [17]. This topographic association among specific sites of the optic disk and their equivalent bundle traces is intricate, with considerable intersubject discrepancy [13,14]. Maxima (thickest) RNFL regions around the disc are known to be located in relation to the locations of the primary blood arteries surrounding the ONH [30]. Blood vessel position was not considered a separate factor in the model. Future research may examine if RNFL distribution and blood vessel position are related to the same ocular factors.

The PES algorithm has certain limitations. Spatial trajectories from retinal regions to the ONH are defined by the crossing of bundle maps with the ONH boundary, which may introduce deviations affecting RNFL architecture and intersubject variability in retinal–ONH associations. To improve accuracy, the algorithm applies two independent methods for enhancing the FO ROI and two for the ONH ROI, each emphasizing contour edges for precise delineation. Because ONH location explains much of the variance in structure–function maps, this step may substantially influence bundle representation [13,14,17]. For comparison, Jansonius et al. [15,16] estimated ONH size using the macula–disc center distance in fundus photographs and defined its position via the papillomacular location, while Lamparter et al. [14] applied magnification-corrected measurements of ONH size and displacement relative to the FO along the longitudinal and latitudinal axes separately. One key element in the PES algorithm is the use of the HFV filter. RNF bifurcations and crossings are one known limitation [43] since local intensity profiles at these sites do not represent a simple, singular vessel-like formation, nor can they be considered isolated formation with uniform intensity characterized solely by cross-sectional curvature and longitudinal orientation. In order to overcome this restriction, (1) the bundle direction is aligned with predefined orientations which removes false positive instances of non-bundle formations, and (2) “group” and “individual” averages are included to guarantee orientation continuity between adjacent extractions. Since this approach has not yet been evaluated, it is unable to detect RNFL deficiencies in early or advanced glaucoma or other ocular atrophy. Future research must concentrate on developing highly reliable methods for identifying ocular neuropathies.

RNFL bundle tracing difficulties could also be a cause of inaccuracy in the final map. The primary limitations in identifying bundle trajectories are relative ocular media opacity in the fundus’ perimeter, low-quality fundus images with low resolution, sharpness, and contrast. High resolution provides the spatial detail necessary to discern the fine, sub-millimeter structures of the RNFL. Low-resolution images can blur the pattern of RNFL axons that typically fan out from the ONH, presenting a faint, semi-transparent striated pattern. This could lead to the loss of subtle textural information critical for identifying and mapping individual fiber bundles. Moreover, low image quality due to poor focus, uneven illumination, overexposure, or underexposure can severely impact the performance of segmentation algorithms, leading to false positive or false negative traces. Image sharpness—a measure of an image’s ability to render fine details with clear, distinct boundaries—is crucial for separating the RNFL bundles from the surroundings. A sharp image minimizes blur, ensuring that the edges of the RNFL bundles are well-defined. This clarity is essential for automated segmentation algorithms to accurately delineate the boundaries of the RNFL tracings, where the HFV filter can alleviate the restriction of RNF bifurcations and crossings. Contrast enhancement is a critical preprocessing step that fundamentally improves the visibility of the RNFL bundles against the reddish-brown retinal background. By selectively amplifying the differences in pixel intensity, it reveals subtle anatomical features that are often obscured by low native contrast, uneven illumination, and noise inherent in the imaging process. RNFL tracing unique texture is more prominent, enabling more reliable segmentation and analysis of its trajectory. The PES algorithm initially applied exclusion criteria to discard fundus images in which the FO and/or OD could not be accurately identified. Additionally, the method mandates a minimum of eleven marker points for fitting the assembled traces with a 10th-order Bézier curve. Computational algorithms provide an effective basis for future empirical testing and experimental hypothesis generation. Computational modeling techniques can benefit from the ease of changing and refining the algorithm in response to innovative empirical data. This flexibility allows for more rigorous validation in subsequent research by allowing the algorithm to keep current with a fast-changing body of information through frequent updates.

## 5. Conclusions

The computational model described in this research is a step toward effectively integrating structural and functional test data from individual patients in the treatment of visual neuropathies like glaucoma. Individually tailored maps connecting every visual field site to the ONH can be produced by feeding the algorithm a few basic biometric data points. This study has also determined a significant impact of ocular characteristics on the eye’s structure-function relationship and highlights the necessity of customized methods for evaluating the association of structure-function data. The quantitative comparisons described here show three findings: (1) the delineation bundle trajectories can be built by image processing methods considering distinct anatomical features that can be tailored to individual eyes; (2) structure–function maps can be designed by image processing methods for control participants; and (3) bundle directional angles can be described by the disk–fovea angle and the ONH location.

## Figures and Tables

**Table 1:** Ocular Parameters of the Study Population.

	ONH_x_ (deg)	ONH_y_ (deg)	ONH–FO Angle (deg)	Ellipticity Ratio	ONH Area (deg^2^)
Mean	16.54	2.07	7.07	0.88	38.45
SD	1.03	1.02	3.64	0.07	6.35
Min	11.57	0.04	−2.60	0.48	22.03
Max	21.11	6.01	19.76	0.99	58.96

**Table 2:** Individual Variation for Given visual field locations (VFL). The mean ± SD (95% limit) of the directional angles at each VF location (2nd and 3rd row, respectively), in degrees. The percentage of variation (PV, 4th row) that is explained by the optimum AIC model.

**VFL**	**2**	**3**	**4**	**5**	**6**	**7**	**8**	**9**	**10**	**12**	**13**	**14**	**15**	**16**	**17**	**18**	**20**
**Mean**	181	186	216	171	176	179	188	212	240	159	160	163	180	211	247	300	149
**SD**	5.0	9.6	16.8	19.7	6.9	5.4	7.3	12.7	17.4	10.9	9.7	9.0	9.9	9.4	10.5	22.8	4.2
**PV**	53.2	67.4	72.8	55.5	65.9	70.4	74.9	73.8	72.7	37.1	30.2	31.4	27.6	45.2	59.6	45.3	75.7
**VFL**	**21**	**22**	**23**	**25**	**30**	**36**	**37**	**38**	**39**	**40**	**41**	**42**	**46**	**47**	**48**	**51**	**52**
**Mean**	146	147	150	198	210	210	208	201	184	148	103	44	160	138	108	157	133
**SD**	7.4	7.3	7.0	6.1	13.9	7.6	6.2	6.2	10.0	9.5	10.3	12.0	7.5	9.5	13.3	9.2	12.6
**PV**	62.5	77.8	57.3	33.1	49.7	41.2	50.3	44.4	43.0	56.1	63.1	60.8	40.8	73.1	69.3	43.9	53.1

**Table 3:** Ocular Parameters of the Study Population. Five ocular parameters and 34 of 52 VF sites entered the analysis with max. 500 intersecting RNFL bundles per site. Parameters in the optimal model are indicated with a cross for each VF site. ‘‘X’’ represents statistical significance (*p* < 0.001).

**VF Location**	**2**	**3**	**4**	**5**	**6**	**7**	**8**	**9**	**10**	**12**	**13**	**14**	**15**	**16**	**17**	**18**	**20**	
**ONH_x_**		X	X	X	X	X	X			X	X	X	X	X	X			
**ONH_y_**		X	X				X		X	X	X		X			X		
**ONH–FO Angle**	X	X	X	X	X	X	X	X	X	X	X	X	X	X	X	X	X	
**Ellipticity**		X						X								X	X	
**ONH area**											X			X		X		
**Total**	1	4	3	2	2	2	3	2	2	3	4	2	3	3	2	4	2	
**VF Location**	**21**	**22**	**23**	**25**	**30**	**36**	**37**	**38**	**39**	**40**	**41**	**42**	**46**	**47**	**48**	**51**	**52**	**Total**
**ONH_x_**	X	X	X	X	X	X	X	X	X	X	X	X	X		X			26
**ONH_y_**	X	X	X		X	X	X	X	X	X	X	X	X		X	X		22
**ONH–FO Angle**	X	X	X	X	X		X	X	X		X	X	X	X	X	X	X	32
**Ellipticity**		X							X									6
**ONH area**		X	X			X	X	X	X									9
**Total**	3	5	4	2	3	3	4	4	5	2	3	3	3	1	3	2	1	

## Data Availability

PES (personalized estimated segmentation) software application developed for the segmentation of nerve fiber trajectories is a code programmed and owned by the author written in MATLAB (R2024b Update 6, version 24.2, 64-bit, Windows; The MathWorks, Inc., Natick, MA, USA), available on GitHub: https://github.com/diegolujv/PES-app.git (accessed on 15 August 2025).

## Abbreviations

The following abbreviations are used in this manuscript:

VF	Visual field
RNFL	Retinal nerve fiber layer
ONH	Optic nerve head
OCT	Optical Coherence Tomography
FO	Fovea
2D	Two-dimensional
3D	Three-dimensional
PES	Personalized estimated segmentation
RNF	Retinal nerve fiber
ROI	Region of interest
CLAHE	Contrast-Limited Adaptive Histogram Equalization
HFV	Hessian-based multiscale Frangi vesselness filter
MM	Maximum-minimum modulation filter
AIC	Akaike information criterion
ROTA	RNFL optical texture analysis

## References

[1] Shan S., Wu J., Cao J., Feng Y., Zhou J., Luo Z., Song P., Rudan I. (2024). Global Health Epidemiology Research Group (GHERG). Global incidence and risk factors for glaucoma: A systematic review and meta-analysis of prospective studies. J. Glob. Health.

[2] Weber J., Ulrich H. (1991). A perimetric nerve fiber bundle map. Int. Ophthalmol..

[3] Weber J., Dannheim F., Dannheim D. (1990). The topographical relationship between optic disc and visual field in glaucoma. Acta Ophthalmol..

[4] Wirtschafter J.D., Becker W.L., Howe J.B., Younge B.R. (1982). Glaucoma visual field analysis by computed profile of nerve fiber function in optic disc sectors. Ophthalmology.

[5] Turpin A., Sampson G.P., McKendrick A.M. (2009). Combining ganglion cell topology and data of patients with glaucoma to determine a structure-function map. Investig. Opthalmol. Vis. Sci..

[6] Rao H.L., Zangwill L.M., Weinreb R.N., Leite M.T., Sample P.A., Medeiros F.A. (2011). Structure-Function Relationship in Glaucoma Using Spectral-Domain Optical Coherence Tomography. Arch. Ophthalmol..

[7] Alluwimi M.S., Swanson W.H., Malik R. (2023). Structure–function assessment in glaucoma based on perimetric sensitivity and en face optical coherence tomography images of retinal nerve fiber bundles. Sci. Rep..

[8] Seeböck P., Vogl W.D., Waldstein S.M., Orlando J.I., Baratsits M., Alten T., Arikan M., Mylonas G., Bogunović H., Schmidt-Erfurth U. (2022). Linking Function and Structure with ReSensNet: Predicting Retinal Sensitivity from OCT using Deep Learning. Ophthalmol. Retin..

[9] Chen Z., Ishikawa H., Wang Y., Wollstein G., Schuman J.S. (2024). Deep-Learning-Based Group Pointwise Spatial Mapping of Structure to Function in Glaucoma. Ophthalmol. Sci..

[10] Chen Z., Shemuelian E., Wollstein G., Wang Y., Ishikawa H., Schuman J.S. (2023). Segmentation-Free OCT-Volume-Based Deep Learning Model Improves Pointwise Visual Field Sensitivity Estimation. Transl. Vis. Sci. Technol..

[11] Park K., Kim J., Lee J. (2020). A deep learning approach to predict visual field using optical coherence tomography. PLoS ONE.

[12] Christopher M., Bowd C., Belghith A., Goldbaum M.H., Weinreb R.N., Fazio M.A., Girkin C.A., Liebmann J.M., Zangwill L.M. (2020). Deep Learning Approaches Predict Glaucomatous Visual Field Damage from OCT Optic Nerve Head En Face Images and Retinal Nerve Fiber Layer Thickness Maps. Ophthalmology.

[13] Garway-Heath D.F., Poinoosawmy D., Fitzke F.W., Hitchings R.A. (2000). Mapping the visual field to the optic disc in normal tension glaucoma eyes. Ophthalmology.

[14] Lamparter J., Russell R.A., Zhu H., Asaoka R., Yamashita T., Ho T., Garway-Heath D.F. (2013). The Influence of Intersubject Variability in Ocular Anatomical Variables on the Mapping of Retinal Locations to the Retinal Nerve Fiber Layer and Optic Nerve Head. Investig. Ophthalmol. Vis. Sci..

[15] Jansonius N.M., Nevalainen J., Selig B., Zangwill L.M., Sample P.A., Budde W.M., Jonas J.B., Lagrèze W.A., Airaksinen P.J., Vonthein R. (2009). A mathematical description of nerve fiber bundle trajectories and their variability in the human retina. Vis. Res..

[16] Jansonius N.M., Schiefer J., Nevalainen J., Paetzold J., Schiefer U. (2012). A mathematical model for describing the retinal nerve fiber bundle trajectories in the human eye: Average course, variability, and influence of refraction, optic disc size and optic disc position. Exp. Eye Res..

[17] Denniss J., Allison M., McKendrick A.T. (2012). An Anatomically Customizable Computational Model Relating the Visual Field to the Optic Nerve Head in Individual Eyes. Investig. Opthalmol. Vis. Sci..

[18] Schiefer U., Flad M., Stumpp F., Malsam A., Paetzold J., Vonthein R., Oliver Denk P., Sample P.A. (2003). Increased detection rate of glaucomatous visual field damage with locally condensed grids: A comparison between fundusoriented perimetry and conventional visual field examination. Arch. Ophthalmol..

[19] Quintana-Quintana O.J., Aceves-Fernández M.A., Pedraza-Ortega J.C., Alfonso-Francia G., Tovar-Arriaga S. (2025). Deep Learning Techniques for Retinal Layer Segmentation to Aid Ocular Disease Diagnosis: A Review. Computers.

[20] Zhang H., Yang B., Li S., Zhang X., Li X., Liu T., Higashita R., Liu J. (2025). Retinal OCT image segmentation with deep learning: A review of advances, datasets, and evaluation metrics. Comput. Med. Imaging Graph..

[21] Luján Villarreal D. (2025). Automatic Algorithm-Aided Segmentation of Retinal Nerve Fibers Using Fundus Photographs. J. Imaging.

[22] Decencière E., Zhang X., Cazuguel G., Lay B., Cochener B., Trone C., Gain P., Ordonez R., Massin P., Erginay A. (2014). Feedback on a publicly distributed database: The Messidor database. Image Anal. Ster..

[23] Zuiderveld K. (1994). Contrast Limited Adaptive Histograph Equalization. Graphic Gems IV.

[24] Frangi A.F., Niessen W.J., Vincken K.L., Viergever M.A. (1998). Multiscale vessel enhancement filtering. Proceedings of the In-ternational Conference on Medical Image Computing and Computer-Assisted Intervention—MICCAI’98.

[25] Burnham K.P., Anderson D.R. (2002). Model Selection and Multimodel Inference: A Practical Information-Theoretic Approach.

[26] Ohad G. (2025). Fit_Ellipse. MATLAB Central File Exchange. https://www.mathworks.com/matlabcentral/fileexchange/3215-fit_ellipse.

[27] Jansonius N.M., Schiefer U. (2020). Anatomical location of the raphe and extended raphe in the human retina: Implications for assessment of the optic nerve with OCT. Trans. Vis. Sci. Tech..

[28] Bikbov M.M., Iakupova E.M., Gilmanshin T.R., Bikbova G.M., Kazakbaeva G.M., Panda-Jonas S., Gilemzianova L.I., Jonas J.B. (2023). Prevalence and associations of nonglaucomatous optic nerve atrophy in high myopia: The ural eye and medical study. Ophthalmology.

[29] Hood D.C., Fortune B., Arthur S.N., Xing D., Salant J.A., Ritch R., Liebmann J.M. (2008). Blood vessel contributions to retinal nerve fiber layer thickness profiles measured with optical coherence tomography. J. Glaucoma.

[30] Hood D.C., Salant J.A., Arthur S.N., Ritch R., Liebmann J.M. (2010). The location of the inferior and superior temporal blood vessels and inter-individual variability of the retinal nerve fiber layer thickness. J. Glaucoma.

[31] Jonas J.B., Jonas R.A., Bikbov M.M., Wang Y.X., Panda-Jonas S. (2023). Myopia: Histology, clinical features, and potential impli-cations for the etiology of axial elongation. Prog. Retin. Eye Res..

[32] Yii F., Gibbon S., MacGillivra T. (2025). Sectoral Changes in Neuroretinal Rim Pallor Across Refractive Error. Ophthalmol. Sci..

[33] Jonas J.B., Xu L., Wei W.B., Pan Z., Yang H., Holbach L., Panda-Jonas S., Wang Y.X. (2016). Retinal Thickness and Axial Length. Investig. Ophthalmol. Vis. Sci..

[34] Fitzgibbon T., Taylor S.F. (1996). Retinotopy of the human retinal nerve fibre layer and optic nerve head. J. Comp. Neurol..

[35] Vrabec F. (1966). The temporal raphe of the human retina. Am. J. Ophthalmol..

[36] Ferreras A., Pablo L.E., Garway-Heath D.F., Fogagnolo P., Garcia-Feijoo J. (2008). Mapping standard automated perimetry to the peripapillary retinal nerve fiber layer in glaucoma. Investig. Ophthalmol. Vis. Sci..

[37] Garvin M.K., Abramoff M.D., Lee K., Niemeijer M., Sonka M., Kwon Y.H. (2012). 2-D pattern of nerve fiber bundles in glaucoma emerging from spectral-domain optical coherence tomography. Investig. Ophthalmol. Vis. Sci..

[38] Wigelius O. (2001). A Model for the Retinal Nerve Fiber Layer. Master’s Thesis.

[39] Leung C.K.S., Lam A.K.N., Weinreb R.N., Garway-Heath D.F., Yu M., Guo P.Y., Chiu V.S.M., Wan K.H.N., Wong M., Wu K.Z. (2022). Diagnostic assessment of glaucoma and non-glaucomatous optic neuropathies via optical texture analysis of the retinal nerve fibre layer. Nat. Biomed. Eng..

[40] Tang L., Scheetz T.E., Mackey D.A., Hewitt A.W., Fingert J.H., Kwon Y.H., Quellec G., Reinhardt J.M., Abràmoff M.D. (2010). Automated quantification of inherited phenotypes from color images: A twin study of the variability of optic nerve head shape. Investig. Opthalmol. Vis. Sci..

[41] Sanfilippo P.G., Cardini A., Hewitt A.W., Crowston J.G., Mackey D.A. (2009). Optic disc morphology–rethinking shape. Prog. Retin. Eye Res..

[42] Hewitt A.W., Poulsen J.P., Alward W.L.M., Bennett S.L., Budde W.M., Cooper R.L., Craig J.E., Fingert J.H., Foster P.J., Garway-Heath D.F. (2007). Heritable features of the optic disc: A novel twin method for determining genetic significance. Investig. Opthalmol. Vis. Sci..

[43] Jerman T., Pernus F., Likar B., Spiclin Z. (2016). Enhancement of Vascular Structures in 3D and 2D Angiographic Images. IEEE Trans. Med. Imaging.

